# Google Trends™ and Quality of Information Analyses of Google™ Searches Pertaining to Concussion

**DOI:** 10.1089/neur.2022.0084

**Published:** 2023-03-24

**Authors:** Mehul Mehra, Pierce A. Brody, Simran Mehrotra, Om Sakhalkar, Todd Maugans

**Affiliations:** ^1^ Department of Neurosurgery, Medical College of Georgia, Augusta University, Augusta, Georgia, USA.; ^2^ College of Osteopathic Medicine, William Carey University, Hattiesburg, Mississippi, USA.

**Keywords:** concussion, Google Trends™, online searches, quality of information, readability

## Abstract

Sports-related concussions occur with high incidence in the United States. Google Trends™ (GT) analyses indicate changes of public interest in a topic over time, and can be correlated with incidence of health events such as concussion. Internet searches represent a primary means of patient education for many health topics, including concussion; however, the quality of medical information yielded by internet searches is variable and frequently of an inappropriate reading level. This study therefore aims to describe GT over time and evaluate the quality and readability of information produced by Google™ searches of the term “concussion.” We identified a strong negative correlation from 2009 to 2016 between GT scores and total number of American high school football participants (*R*^2^ = 0.8553) and participants per school (*R*^2^ = 0.9533). Between 2004 and 2020, the monthly GT popularity score were variable (*p* = 3.193E-08), with September having the greatest scores, correlating with the height of American tackle football season. Applying five validated quality assessment scoring systems at two time points, it was confirmed that different sources yielded varying quality of information. Academic and non-profit healthcare sources demonstrated the highest quality metrics across two time points. There was significant variability of scores among the different scoring systems, however. The majority of searches at both time points yielded information that was rated as “fair” to “poor” in quality. Applying six readability tests, we revealed that only a single commercial website offered information written at or below the American Medical Association– recommended 6th-grade level for healthcare information. In summary, GT data analyses suggest that searches correlate with the American tackle football season and increased between 2009 and 2016, given that public interest in concussion increased and annual participation in football decreased. The quality of information yielded by Google™ searches and readability are inadequate, indicating the need for significant improvement.

## Introduction

Concussion has the highest incidence of traumatic brain injuries (TBIs) and represents a major public health concern. Sports-related concussions have received much attention in the medical and lay press over the past two decades. Contact-collision sports have the highest rates of concussion and are played seasonally.

The internet is used commonly to research health and medical topics; however, the veracity of information derived from such searches has been found to vary considerably. Based on ubiquitous access to the internet, it is logical to assume that the internet is used by patients and family members to self-educate after a TBI has occurred.

In an effort to understand how the internet is used in researching concussion, we set out to study trends in internet use.

When studying trends, both infodemiology and infovelliance provide valuable insight. This type of data is already used to monitor a variety of searches, including disease outbreaks, through popular websites like Twitter and Google™. Launched in 2006, Google Trends™ (GT) is a tool that analyzes trends in search queries based on public interest and popularity.^1^ Trillions of searches take place on Google annually, translating to several billion searches in a day. At least 90% of searches around the world take place on Google. GT provides real-time and archived information regarding Google searches from 2004 onward, and it allows users to choose based on geographical region, year, time span, and many other categories. It shows changes in interest in a given time period in any country or region for a selected term. GT data can be adjusted by year as well as by comparison between the same month across several years by using a variety of filters, such as Trending Searches, Year in Search, and Explore. The database takes into account spelling mistakes, accents, and plurality forms, ensuring a valid analysis.

Google searches have a strong correlation with current events, health and medical searches being most focused. Public reaction toward healthcare information can also be detected from GT and has been especially useful in recent outbreaks and epidemics.^2,3^ Thus, we hypothesize that GT can be used to study concussion frequency and rates, because it may show relationships between internet searches and changes in year-over-year and seasonal rates of sports participation.

We also aimed to study the veracity of information provided by internet searches using the search term “concussion.” Sources of information available on the internet are myriad and can profoundly affect the quality of information. Broad categories include personal, professional, organizational, commercial, governmental, and educational. It is logical to assume that websites affiliated with “authoritative” entries, such as education and governmental organizations, will provide the most accurate and germane information; however, that assumption should be tested.

There are many tools used to objectively rate quality of information on these websites. DISCERN is an instrument that helps users determine the quality of treatment choices that the website is providing through a series of questions.^4^ The HonCode Principles is one the earliest sets of quality markers for evaluating online healthcare information.^5^ JAMA Benchmarks are a set of four criteria that have been set by the American Medical Association (AMA), and criteria are based on Authorship, Attribution, Currency, and Disclosure.^6^ The Currency, Relevance, Authority, Accuracy, and Purpose (CRAAP) test was developed by the senior author of this article as a more comprehensive evaluation tool, with a set of criteria for each section.^7^

Finally, readability is germane to knowledge acquisition. The AMA recommends that material should be written at a 6th-grade reading level to maximize understanding by the broadest segment of the U.S. population. We have chosen to use several automated scoring programs to ascertain the accessibility of information derived from internet searches.

## Methods

### Google Trends™

For individual search queries, GT displays the relative search volume (RSV), which is proportionate to the searches for that query over time, rather than the absolute search quantity. RSV is displayed as scores 0–100. The score is a normalized proportion of a specific query's search volume divided by the total searches for all queries in a time period, geographical location, and topic filter. This means that the score of 0 is very few searches rather than none. Moreover, if the scores are the same between two different geographical locations or two different time points, the absolute search quantity may not be the same, but the normalized proportions are equivalent.

We performed our GT search on April 30, 2021, using the *Injury* query of “concussion” with the categorical filter “Health” from 2004 to the present using the type of search “web search” and location “United States.” The *Injury* topic was used instead of the *search term* because our search was not concerned with searches unrelated to the concussion injury, such as searches intended for the 2015 film *Concussion*. Because American tackle football results in the greatest annual incidence of concussions, we aimed to ascertain whether GT data could be correlated with U.S. high school football participation. We derived appropriate statistics from the National Federation of State High School Associations' High School Participation Survey Archive. The GT analysis was replicated by reacquiring the data and reanalyzing immediately after the analysis and after figure production to ensure accuracy and reproducibility.

### Quality of internet information

This study limited websites to the first page of Google searches because most seekers of health information do not go past the first page.^8^ Two searches were performed in the incognito mode on August 13, 2021 with the quality analysis performed between August 13 and August 15, 2021 and on June 16, 2022 with the analysis performed between June 16 and June 19, 2022. Two searches were conducted to assess possible differences in scoring over time. Exclusion criteria included PDF documents, videos, URLs labeled as advertisements, and PowerPoint presentations.

Two independent reviewers assessed the quality of each website independently by website type. The following validated quality assessments were used: DISCERN; Health-on-The-Net (HON) Foundation code and certification status; JAMA Benchmarks; CRAAP test; and treatment content. See Appendix 1 for details of these tools. Website type was separated by broad categories: government; commercial; and academic, non-profit healthcare sources.

### Readability analysis

Each website assessed for quality of information was also analyzed for readability. According to the AMA, healthcare material should not be written above a 6th-grade reading level.^9^ Six readability tests were applied: Flesch-Kincaid Reading Ease (FKRE); Flesch-Kincaid Grade Level (FKGL); Gunning Fog Index (GFI); Coleman-Liau Index (CLI); Simple Measure of Gobbledygook Index (SMOG); and Automated Readability Index (ARI). The analysis was performed with an automated analyzer available online by pasting each website's article text only into the tool (see Appendix 2).

This study was considered exempt by institutional review board review because we examined publicly available, deidentified secondary data throughout the completed GT, Quality of Internet Information, and Readability analyses.

### Statistical analysis

One-way analysis of variance (ANOVA) with α = 0.05 was performed on GT RSV scores to determine the presence of a statistically significant month-specific seasonal search pattern. Pearson's correlation coefficients were determined between average yearly GT scores for “concussion” and annual high school participation in football and all sports obtained from the National Federation of State High School Associations. Descriptive statistics were used for each website and website classifications for the quality-of-information analysis. Cohen's kappa test was used to assess inter-rater reliability (two reviewers) for DISCERN, HONcode, JAMA Benchmarks, CRAAP test, and treatment score. Cohen's kappa scores were classified as excellent, good, fair, and poor for 1.00–0.75, 0.74–0.60, 0.59–0.40, and <0.4, respectively.^10^ Subsequently, raters then engaged in discussion to rectify each discrepancy in scoring to determine consensus scores. Concern for a possible non-normal distribution of consensus scores led to the decision to produce descriptive statistics only. Data were analyzed using Microsoft Excel (version 16.65; Microsoft Corporation, Redmond, WA) and SPSS software (version 27; IBM Corp, Armonk, NY).

## Results

### Google Trends™

**FIG. 1.**
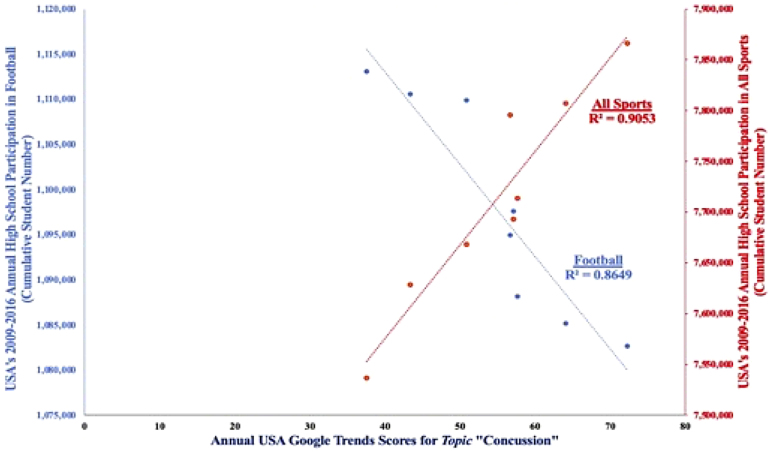
Association of concussion Google Trends™ scores with the U.S. 2009–2016 annual high school participation in all sports and football. Pearson's correlation coefficient was calculated in Microsoft Excel (Microsoft Corporation, Redmond, CA) between the average yearly Google Trends™ scores for “concussion” and yearly participation data derived from the National Federation of State High School Associations. Results show that football participation has a strong negative correlation with Google Trends™ scores whereas all sports has a strong positive correlation.

**FIG. 2.**
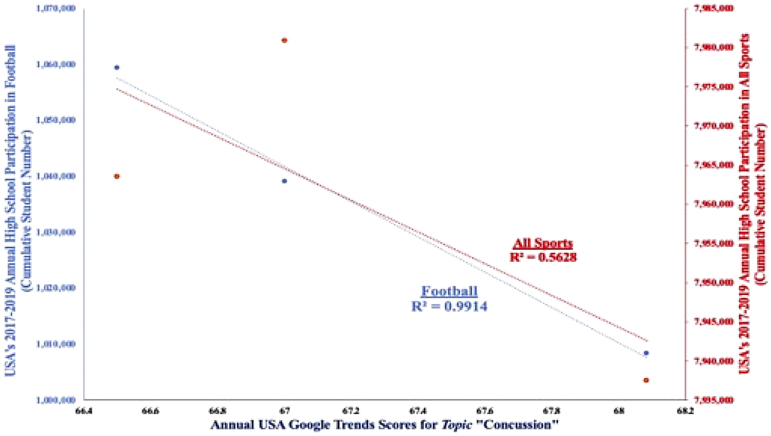
Association of concussion Google Trends™ scores with the U.S. 2017–2019 annual high school participation in all sports and football. Pearson's correlation coefficient was calculated in Microsoft Excel (Microsoft Corporation, Redmond, CA) between the average yearly Google Trends™ scores for “concussion” and yearly participation data derived from the National Federation of State High School Associations. Results show that football participation has a strong negative correlation with Google Trends™ scores.

**FIG. 3.**
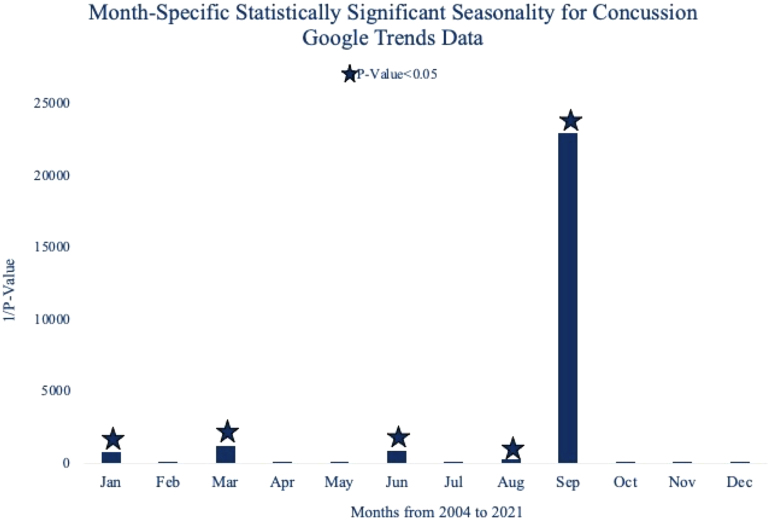
Month-specific statistically significant seasonality of Google Trends™ data. Monthly variability calculated with one-way ANOVA regression analysis from 2004 to 2021 with α = 0.05 using Microsoft Excel (Microsoft Corporation, Redmond, WA). January, March, June, August, and September had statistically significant seasonality over the time period analyzed, suggesting that certain months had seasonally increased or decreased search volumes. ANOVA, analysis of variance.

**FIG. 4.**
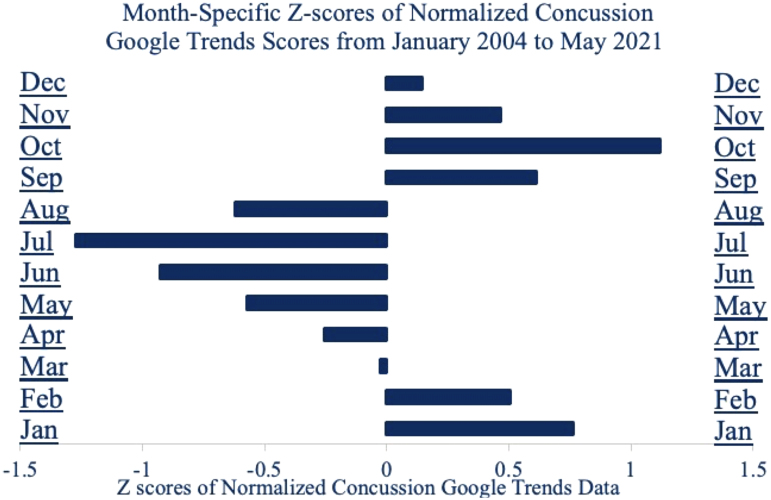
Month-specific z-scores of normalized concussion Google Trends™ scores from January 2004 to May 2021. Average monthly z-scores were calculated in Microsoft Excel (Microsoft Corporation, Redmond, CA). Fall and winter months, September through February, showed above-average z-scores whereas the spring and summer months, March through August, showed below-average z-scores, showing that there is more public interest in concussion in the fall and winter months.

The RSV began to rise after 2009 and eventually peaked in December 2015 (100 of 100). The RSV reached a minimum in April 2004 (9 of 100). Visually, the RSV displayed a yearly peaked search interest in the late summer and early fall months. Additionally, scores fell substantially in the months after the onset of the COVID-19 Pandemic. Pearson's correlation coefficients demonstrated strong negative correlation from 2009 to 2016 between RSVs for concussion with the total number of American high school football participants (*R*^2^ = 0.8553), whereas there was a positive correlation with all high school sports participants (*R*^2^ = 0.9053; Fig. 1). Subsequently, from 2017 to 2019, there was a strong negative correlation between RSVs for concussion with the total number of high school football participants (*R*^2^ = 0.9914), whereas there was a weak correlation with all high school sports participants (*R*^2^ = 0.5628; Fig. 2). Confirming football-related seasonality, one-way ANOVA regression analysis concluded that the 2004 to the 2020s mean change in month-specific popularity score is not the same (*p* = 3.193E-08), and the months during the high school football season had statistically significant variability (September: *p* = 4.389E-05) with elevated average z-scores (Figs. 3 and 4).

### Quality of information

Nine results were displayed on the first page of Google on both time points of August 13, 2021 and June 15, 2022. Cohen's kappa inter-rater reliability was excellent for each scoring metric (Tables 1 and 2). The website categories government; academic, non-profit healthcare source; and commercial each had three websites during the first analysis and two, five, and two, respectively, in the second analysis. Among the website categories in the first analysis, academic, non-profit healthcare had the highest means for all scoring tools except for the treatment score, which was highest in the commercial category. In the second analysis, academic, non-profit healthcare had the highest means for the scoring tools DISCERN, HONcode, and Treatment. The commercial category had the highest mean for JAMA Benchmarks, and government had the highest mean for CRAAP. The government category had the lowest scores for each tool except for CRAAP in both analyses (Tables 3 and 4).

**Table 1.** Cohen's Kappa Inter-Rater Reliability Overall and by Website for 2021 “Concussion” Google™ Search Results

Website no.	Website hyperlink	DISCERN	CRAAP	HonCode	JAMA Benchmarks	Treatment scores
1	https://www.cdc.gov/headsup/basics/concussion_whatis.html#:~:text=A%20concussion%20is%20a%20type,move%20rapidly%20back%20and%20forth	1.00	0.93	1.00	1.00	1.00
2	https://www.mayoclinic.org/diseases-conditions/concussion/symptoms-causes/syc-20355594	0.75	1.00	1.00	1.00	1.00
3	https://www.cdc.gov/headsup/basics/concussion_symptoms.html	0.82	1.00	1.00	1.00	1.00
4	https://www.webmd.com/brain/concussion-traumatic-brain-injury-symptoms-causes-treatments	0.84	0.93	1.00	1.00	1.00
5	https://www.aans.org/en/Patients/Neurosurgical-Conditions-and-Treatments/Concussion	0.82	1.00	1.00	1.00	1.00
6	https://medlineplus.gov/concussion.html	1.00	0.86	1.00	1.00	1.00
7	https://orthoinfo.aaos.org/en/diseases--conditions/sports-concussion	0.91	1.00	1.00	1.00	1.00
8	https://www.healthline.com/health/concussion	0.81	1.00	1.00	1.00	1.00
9	https://kidshealth.org/en/parents/concussions.html	1.00	1.00	1.00	1.00	1.00
All	All	0.89	0.97	1.00	1.00	1.00


Kappa results of 0 indicate no agreement, 0.01–0.20 as none to slight, 0.21–0.40 as fair, 0.41–0.60 as moderate, 0.61–0.80 as substantial, and 0.81–1.00 as almost perfect agreement.

CRAAP, Currency, Relevance, Authority, Accuracy, and Purpose; HON, Health-on-The-Net.

**Table 2.** Cohen's Kappa Inter-Rater Reliability Overall and by Website for 2022 “Concussion” Google™ Search Results

Website no.	Website hyperlink	DISCERN	CRAAP	HonCode	JAMA Benchmarks	Treatment scores
1	https://www.mayoclinic.org/diseases-conditions/concussion/symptoms-causes/syc-20355594#:~:text=A%20concussion%20is%20a%20traumatic%20blow%20to%20the%20head	0.84	1.00	1.00	1.00	1.00
2	https://www.cdc.gov/headsup/basics/concussion_whatis.html	1.00	0.93	1.00	1.00	1.00
3	https://www.webmd.com/brain/concussion-traumatic-brain-injury-symptoms-causes-treatments	0.84	0.93	1.00	1.00	1.00
4	https://www.aans.org/en/Patients/Neurosurgical-Conditions-and-Treatments/Concussion	0.82	1.00	1.00	1.00	1.00
5	https://en.wikipedia.org/wiki/Concussion	0.83	0.92	1.00	1.00	1.00
6	https://www.healthline.com/health/concussion	0.90	1.00	1.00	1.00	1.00
7	https://medlineplus.gov/concussion.html	1.00	0.86	1.00	1.00	1.00
8	https://concussionfoundation.org/concussion-resources/what-is-concussion	0.87	0.87	1.00	1.00	1.00
9	https://www.hopkinsmedicine.org/health/conditions-and-diseases/concussion	1.00	1.00	1.00	1.00	1.00
All	All	0.91	0.95	1.00	1.00	1.00

Kappa results of 0 indicate no agreement, 0.01–0.20 as none to slight, 0.21–0.40 as fair, 0.41–0.60 as moderate, 0.61–0.80 as substantial, and 0.81–1.00 as almost perfect agreement.

CRAAP, Currency, Relevance, Authority, Accuracy, and Purpose; HON, Health-on-The-Net.

**Table 3.** “Concussion” Google™ Search Website Quality, Treatment, and Readability Scores and Statistics for 2021, Overall and by Website Categories

All websites	DISCERN	CRAAP	HonCode	JAMA Benchmarks	Treatment guidelines	Flesch-Kincaid Reading Ease score	Flesch-Kincaid Grade Level	Gunning-Fog Index	Coleman-Liau Index	SMOG Index	Automated Readability Index
Mean	37	27.33	5.89	2.78	2.56	58.52	8.09	10.87	10.68	11.44	7.91
Standard deviation	8.38	2.06	1.05	.67	1.67	9.35	1.39	1.41	1.89	1.18	1.63
Range	29–57	24–30	4–7	2–4	0–6	N/A	N/A	N/A	N/A	N/A	N/A
Mean percentage of maximum	46.25	80.39	73.61	69.44	25.56						

Government websites	DISCERN	CRAAP	HonCode	JAMA Benchmarks	Treatment Guidelines	Flesch-Kincaid Reading Ease score	Flesch-Kincaid Grade Level	Gunning-Fog Index	Coleman-Liau Index	SMOG Index	Automated Readability Index
Mean	32.33	26.67	5.33	2.00	1.33	65.83	7.50	10.63	8.97	11.53	7.13
Standard deviation	2.89	1.53	1.53	0.00	1.15	3.25	1.23	1.86	0.23	1.57	1.27
Range	29–34	25–28	4–7	2–2	0–2	N/A	N/A	N/A	N/A	N/A	N/A
Mean percentage of maximum	40.42	78.43	66.67	50.00	13.33						

Academic websites	DISCERN	CRAAP	HonCode	JAMA Benchmarks	Treatment guidelines	Flesch-Kincaid Reading Ease score	Flesch-Kincaid Grade Level	Gunning-Fog Index	Coleman-Liau Index	SMOG Index	Automated Readability Index
Mean	41.33	29.33	6.67	3.33	2.67	47.93	9.50	11.87	12.90	12.17	9.67
Standard deviation	13.65	1.15	0.58	0.58	1.15	1.91	0.79	0.71	0.46	0.93	1.10
Range	32–57	28–30	6–7	3–4	2–4	N/A	N/A	N/A	N/A	N/A	N/A
Mean percentage of maximum	51.67	86.27	83.33	83.33	26.67						

General websites	DISCERN	CRAAP	HonCode	JAMA Benchmarks	Treatment guidelines	Flesch-Kincaid Reading Ease score	Flesch-Kincaid Grade Level	Gunning-Fog Index	Coleman-Liau Index	SMOG Index	Automated Readability Index
Mean	37.33	26.00	5.67	3.00	3.67	61.80	7.27	10.10	10.17	10.63	6.93
Standard deviation	5.03	2.00	0.58	0.00	2.08	8.41	1.01	1.23	1.37	0.65	0.90
Range	32–42	24–28	5–6	3–3	2–6	N/A	N/A	N/A	N/A	N/A	N/A
Mean percentage of maximum	46.67	76.47	70.83	75.00	36.67						

Websites without HonCode	DISCERN	CRAAP	HonCode	JAMA Benchmarks	Treatment guidelines	Flesch-Kincaid Reading Ease score	Flesch-Kincaid Grade Level	Gunning-Fog Index	Coleman-Liau Index	SMOG Index	Automated Readability Index
Mean	33.67	27.67	6.00	2.67	2.67	60.42	8.10	10.93	10.30	11.67	7.98
Standard deviation	3.01	1.63	1.26	0.82	2.07	10.92	1.71	1.66	2.22	1.41	2.02
Range	29–38	25–30	4–7	2–4	0–6	N/A	N/A	N/A	N/A	N/A	N/A
Mean percentage of maximum	42.08	81.37	75.00	66.67	26.67						

Websites with HonCode	DISCERN	CRAAP	HonCode	JAMA Benchmarks	Treatment guidelines	Flesch-Kincaid Reading Ease score	Flesch-Kincaid Grade Level	Gunning-Fog Index	Coleman-Liau Index	SMOG Index	Automated Readability Index
Mean	43.67	26.67	5.67	3.00	2.33	54.73	8.07	10.73	11.43	11.00	7.77
Standard deviation	12.58	3.06	0.58	0.00	0.58	4.38	0.61	1.00	0.85	0.36	0.55
Range	32–57	24–30	5–6	3–3	2–3	N/A	N/A	N/A	N/A	N/A	N/A
Mean percentage of maximum	54.58	78.43	70.83	75.00	23.33						


Quality scoring tools listed are DISCERN, HONcode and certification status, JAMA Benchmarks, CRAAP test, and Treatment score. Readability formulas listed are Flesch-Kincaid Reading Ease, Flesch-Kincaid Grade Level, Gunning-Fog Index, Coleman-Liau Index, SMOG Index, and Automated Readability Index. Website categories used were Government, Commercial Health Information, and Academic/Nonprofit/Healthcare Providers, each with three websites.

CRAAP, Currency, Relevance, Authority, Accuracy, and Purpose; HON, Health-on-The-Net; SMOG, Simple Measure of Gobbledygook Index; N/A, not applicable.

**Table 4.** “Concussion” Google™ Search Website Quality, Treatment, and Readability Scores and Statistics for 2022, Overall and by Website Categories

All Websites	DISCERN	CRAAP	HonCode	JAMA Benchmarks	Treatment guidelines	Flesch-Kincaid Reading Ease score	Flesch-Kincaid Grade Level	Gunning-Fog Index	Coleman-Liau Index	SMOG Index	Automated Readability Index
Mean	36.78	25.22	5.78	2.33	2.56	54.97	8.09	10.87	10.68	11.44	7.91
Standard deviation	11.68	4.15	1.09	0.71	1.42	10.14	1.39	1.41	1.89	1.18	1.63
Range	24–57	19–30	4–7	1–3	0–5	36.8–67.9	6.7–12.1	9.6–15.0	9.0–14.3	9.3–14.6	6.2–12.3
Mean percentage of maximum	45.97	74.18	72.22	58.33	25.56						

Government websites	DISCERN	CRAAP	HonCode	JAMA Benchmarks	Treatment guidelines	Flesch-Kincaid Reading Ease score	Flesch-Kincaid Grade Level	Gunning-Fog Index	Coleman-Liau Index	SMOG Index	Automated Readability Index
Mean	31.50	26.00	5.50	2.00	1.00	58.75	7.80	10.60	10.75	11.05	7.65
Standard deviation	3.54	2.83	2.12	0.00	1.41	12.23	1.13	0.71	2.33	0.07	1.06
Range	29–34	24–28	4–7	2–2	0–2	50.1–67.4	7.0–8.6	10.1–11.1	9.1–12.4	11.0–11.1	6.9–8.4
Mean percentage of maximum	39.38	76.47	68.75	50.00	10.00						

Academic websites	DISCERN	CRAAP	HonCode	JAMA Benchmarks	Treatment guidelines	Flesch-Kincaid Reading Ease score	Flesch-Kincaid Grade Level	Gunning-Fog Index	Coleman-Liau Index	SMOG Index	Automated Readability Index
Mean	38.40	24.80	6	2.20	3.20	52.88	9.38	12.26	11.58	12.06	9.72
Standard deviation	15.47	5.54	1	0.84	1.31	12.32	1.94	1.90	2.40	2.19	2.42
Range	24–57	19–30	5–7	1–3	2–5	36.8–67.9	6.7–12.1	9.7–15.0	9.0–14.3	9.3–14.6	6.2–12.3
Mean percentage of maximum	48.00	72.94	75.00	55.00	32.00						

Commercial websites	DISCERN	CRAAP	HonCode	JAMA Benchmarks	Treatment guidelines	Flesch-Kincaid Reading Ease score	Flesch-Kincaid Grade Level	Gunning-Fog Index	Coleman-Liau Index	SMOG Index	Automated Readability Index
Mean	38.00	25.50	5.5	3.00	2.50	56.40	7.85	10.35	11.30	10.90	7.70
Standard deviation	7.07	2.12	0.71	0.00	0.71	3.39	0.64	1.06	0.28	0.42	0.42
Range	33–43	24–27	5–6	3–3	2–3	54.0–58.8	7.4–8.3	9.6–11.1	11.1–11.5	10.6–11.2	7.4–8.0
Mean percentage of maximum	47.50	75.00	68.75	75.00	25.00						

Websites without HonCode	DISCERN	CRAAP	HonCode	JAMA Benchmarks	Treatment guidelines	Flesch-Kincaid Reading Ease score	Flesch-Kincaid Grade Level	Gunning-Fog Index	Coleman-Liau Index	SMOG Index	Automated Readability Index
Mean	33.00	24.33	5.83	2.00	2.67	53.30	8.93	11.63	11.72	11.52	9.23
Standard deviation	10.43	4.59	1.33	0.63	1.75	12.17	1.99	1.89	2.17	1.90	2.46
Range	24–52	19–30	4–7	1–3	0–5	36.8–67.9	6.7–12.1	9.7–15.0	9.0–14.3	9.3–14.6	6.2–12.3
Mean percentage of maximum	41.25	71.57	72.92	50.00	26.67						

Websites with HonCode	DISCERN	CRAAP	HonCode	JAMA Benchmarks	Treatment guidelines	Flesch-Kincaid Reading Ease score	Flesch-Kincaid Grade Level	Gunning-Fog Index	Coleman-Liau Index	SMOG Index	Automated Readability Index
Mean	44.33	27.00	5.67	3.00	2.33	58.30	8.20	11.13	10.57	11.70	7.97
Standard deviation	12.06	3.00	0.58	0.00	0.58	4.07	0.75	1.55	1.29	1.42	0.55
Range	33–57	24–30	5–6	3–3	2–3	54.0–62.1	7.4–8.9	9.6–12.7	9.1–11.5	10.6–13.3	7.4– 8.5
Mean percentage of maximum	55.42	79.41	70.83	75.00	23.33						

Quality scoring tools listed are DISCERN, HONcode and certification status, JAMA Benchmarks, CRAAP test, and Treatment score. Readability formulas listed are Flesch-Kincaid Reading Ease, Flesch-Kincaid Grade Level, Gunning-Fog Index, Coleman-Liau Index, SMOG Index, and Automated Readability Index. Website categories used were Government (2), Commercial Health Information (2), Academic/Nonprofit/Healthcare Providers (5), websites with the HonCode Logo (3), and websites without the HonCode Logo (6).

CRAAP, Currency, Relevance, Authority, Accuracy, and Purpose; HON, Health-on-The-Net; SMOG, Simple Measure of Gobbledygook Index; N/A, not applicable.

There were three websites that displayed the HONcode logo in each analysis. In the first analysis, mean scores for DISCERN and JAMA Benchmarks were higher among websites that displayed the logo whereas mean scores for CRAAP, HONcode, and Treatment scores were higher among sites that did have the logo (Table 1). In the second analysis, mean scores for DISCERN, JAMA Benchmarks, and CRAAP were higher among websites that did display the logo whereas mean scores for HONcode and Treatment scores were higher among sites that did not display the logo (Table 4).

The Treatment score mean for all websites was 28% in the first analysis and 26% in the second. The most commonly listed treatments were Physical Rest and Cognitive Rest in both analyses. Aerobic Treatment, Vestibulo-Oculomotor Dysfunction Management/Treatment, and Psychosocial/Emotional Support were not listed by any website in either analysis whereas Cognitive Impairment Management/Treatment and Sleep Management/Treatment were listed only by one website each.

### Readability

Readability was calculated for the same nine results in each analysis. All of the metrics have numerical outputs that indicate higher readability for lower outputs except for FKRE, which has a numerical output that indicates higher readability for higher outputs. When comparing mean scores of website categories, in both analyses the academic/non-profit/healthcare provider category's mean scores were highest for all metrics but FKRE, which was the lowest (Tables 3 and 4). The commercial category's mean scores were lowest for FKGL, GFI, SMOG, and ARI in the first analysis and were lowest in GFI and SMOG in the second analysis. The government category's mean scores were lowest for CLI and highest for FKRE in the first analysis. The government category's mean scores were highest for FKRE and lowest for FKGL, CLI, and ARI. Two websites using FKGL and three websites using ARI were at or under the recommended 6th-grade level in the first analysis. One website using FKGL and two websites using ARI were at or under the recommended level in the second analysis. No other metric ever scored at or under the recommended level in either analysis.

## Discussion

### Google Trends™

Our results indicate that search interest for concussions increases during the American football season in the United States. Previous studies have linked diagnosis with specific medical conditions, such as osteoarthritis, to a high likelihood of using the internet to research the condition.^12^ Additionally, these patients are more likely to engage actively in their care with their physician after online research by asking questions and exploring additional treatment options.^12^ This highlights the importance of patient internet searches on patient self-education and the need for research into condition-specific search patterns.

The rise in RSV in the months during the youth football season highlighted that an increase in concern or incidence of concussion may be associated with youth football. Though this study was limited to correlative relationships and could not determine causality, when annual high school participation in sports was compared to the annual average RSV from 2009 to 2019, we found that as average RSV increased, there was a correlating concomitant drop in high school football participation that did not occur in other sport categories regardless of the months in their respective seasons.^13^ This suggests that youth football concussions or concern for concussions may have been a major driver for RSV seasonality that we observed.

Interestingly, a spike in RSV was found from November 2015 to January 2016, coinciding with the release of the movie *Concussion* on November 10, 2015. This suggests that internet searches may be driven by causes other than concern over the individual injury, such as developments in popular culture.

### Quality of information

We ascertained that there was a high degree of variability in the quality of information yielded by the Google searches. Further, there was significant variance among the scoring systems applied to the search results. For example, using the average scores of the first analysis, the average score was 46% of the total possible for DISCERN despite being 80% of the total possible for CRAAP. This asymmetry highlights the importance of using multiple tools to fully evaluate an online resource. We found that academic, non-profit healthcare sources' average scores were the highest in most quality metrics in both time-point analyses, indicating that websites in this category were the best sources of higher quality information.

There are confounding results, however. Despite having the highest DISCERN average in both analyses, the average DISCERN score for the academic, non-profit healthcare sources was found to be classified only as “fair” in the first analysis and “poor” in the second. The average score overall for each analysis was classified as “poor” also. This indicates that the aggregate information available from Google searches is far from optimal quality, whereas the similarly poor scores between the two time points demonstrates a lack of improvement over time.

Finally, HONcode certification seemed to have little relevance to scores given that the average score for websites displaying the logo were only higher in three of the five categories in the first analysis and two of five in the second. A previous study for online concussion information and readability also found that a majority of websites in 2012 also did not have the logo.^2^

### Readability

Many articles have shown that online healthcare educational information most frequently does not meet the recommended levels for the general public using a variety of scoring systems.^2,3,5^ This study reaches the same conclusion about health information regarding concussions. When assessing individual search results, only 8 of 108 results meet the desired grade level across the two time-point analyses. Only two websites achieved two scores at the 6th-grade level (FKGL and ARI). A 2012 concussion information study revealed the same poor readability results, highlighting the lack in progress over the past decade.^2^

In summary, our study revealed that GT analyses provide insight into the timing and frequency of searches pertaining to concussion. These correlated most strongly with seasonality of American tackle football and varied over the years based on the level of public interest in this topic. Overall, the first-page results revealed by Google searches of concussion yielded websites that offered a variable, but overall suboptimal, quality of information. Further, readability of the information associated with these websites was frequently at a higher educational grade level than recommended by the AMA. This indicates the need to improve the quality and readability of Google search-derived information pertaining to concussion.

## Transparency, Rigor, and Reproducibility Summary

The study was not eligible for clinical trial registration or institutional review board review.^1^ The planned analysis was not formally pre-registered, but the lead authors certify that the analysis plan was pre-specified.^2^ There was a sample size of 210 monthly GT RSVs, 11 years of U.S. high school sports enrollment data, and 18 websites. GT RSVs were analyzed with correlation coefficients and ANOVA analysis of seasonality with *p* < 0.05. All 18 websites were screened with exclusion criteria, with analysis limited to descriptive statistics.^3^

No inconsistencies in data availability or quality were discovered to justify data exclusion.^4^ There were no participants who required blinding. Data analyses were performed by investigators blinded to the sport that GT data were compared with, but not the time the data reflected. During quality evaluation, two raters scored websites separately to limit bias, whereas rater agreement and summary statistics were performed by a third investigator blinded to score-specific rater identity.^5^ Data were acquired between April 30, 2021 and June 19, 2022 between 8:00 am and 9:00 pm, using computers with internet access and incognito Google search. Repeat data characteristics acquisition occurred in 10% of attempts because of user error. Data were analyzed using Microsoft Excel version 16.65 and SPSS version 27. Data were analyzed in three groups: GT RSVs, 2021 quality ratings, and 2022 quality ratings, with 0% batch analysis failure.

After appropriate data acquisition, no unexpected events occurred during the study.^6^ All equipment and software used are widely available from Apple, Microsoft, and IBM. The GT RSVs, sports enrollment data, website links, and quality scoring system rubric links are available from the authors and openly accessible.^7^ Key inclusion criteria are established-standards, internet-derived data research, with methodology validation including other studies analyzing Google-derived data reliability and insights such as Rovetta and colleagues (*Frontiers*) and Nuti and colleagues (*PLoS One*). Future validation will require further clinical correlation, and the primary clinical outcome's test-retest reliability is not formally determined. Key inclusion criteria and clinical outcomes were reviewed by an investigator with previous experience using Google-derived data and pediatric neurosurgery board certification.^9^ Statistical tests were based on the assumption of GT self-reported normalized data sets. Potential non-normal distribution of quality ratings and low sample size limited their analysis to descriptive statistics. Sample sizes and degrees of freedom reflect the number of independent measurements. Data characteristics review addressed any non-independence of measurements.

No missing data were noted. Data sources were not appropriate for reporting primary outcome effect sizes and confidence intervals. Statistical analysis and/or review was performed by a pediatric neurosurgeon and medical students with statistical course training in Microsoft Office and SPSS.^10^ Methods used do not require correction for multiple comparisons, with original and corrected measures of statistical error rates reported in the text.^11^ This report includes documentation of internal replication for GT RSV analysis. Internal quality rating replication is ongoing.^12^ Data are available online and incognito Google search of “concussion.”^13^ Analytical codes used are available only from the authors.^1^

## Abbreviations Used

AMA: American Medical Association
ANOVA: analysis of variance
ARI: Automated Readability Index
CLI: Coleman-Liau Index
CRAAP: Currency, Relevance, Authority, Accuracy, and Purpose
FKGL: Flesch-Kincaid Grade Level
FKRE: Flesch-Kincaid Reading Ease
GFI: Gunning Fog Index
GT: Google Trends™
HON: Health-on-The-Net
RSV: relative search volume
SMOG: Simple Measure of Gobbledygook Index
TBI: traumatic brain injury

## Appendix

### Appendix 1

#### Quality assessment tools

The DISCERN instrument is the first standardized tool for assessing health information available online.^4^ It consists of 16 questions each scored 1–5, with 1 being an absolute NO, 5 being an absolute YES, and 2–4 for websites that partially satisfy the question. Further, the websites are put into five classifications based on DISCERN score: excellent (63–75), good (51–62), fair (39–50), poor (27–38), and very poor (15–26).

The HONcode is an ethical code developed by the HON Foundation and supported by the World Health Organization comprising eight values to promote ethical and reliable information online.^14^ Websites can also apply to be certified by the organization and display the HON logo based on evaluation.

JAMA Benchmarks are a set of four criteria (Authorship, Attribution, Disclosure, and Currency) from the Journal of the American Medical Association.^6^

The CRAAP test is a tool used to assess reliability of information across academic disciplines and across media formats. The modified version used in this study is a 23-question scoring scale that has a higher readability than other instruments and was originally modified in a quality-of-information study in 2018.^7^

The treatment score was developed in order to assess the specific quality of information on concussion treatments because the previous tools do not assess the accuracy of treatment content. The score was developed from the Centers for Disease Control and Prevention Guideline on the Diagnosis and Management of Mild Traumatic Brain Injury Among Children, where 1 point was given for each treatment on a webpage that was also mentioned in the guidelines.^15^

### Appendix 2

#### Readability scoring systems

The FKRE and FKGL are both formulas based on the average sentence length and the average syllables per word to determine readability.^16,17^ The GFI formula is based on average sentence length and the proportion of polysyllabic words (three or more syllables) to all words.^18^ The CLI is unique from the previous two in that it does not take into account the syllables. It is instead calculated based on the average number of letters and number of sentences per 100 words.^19^ The SMOG is based on samples of 10 sentences from the beginning, middle, and end of a text and calculating the square root of the total number of polysyllabic words.^20^ The ARI relies on the average number of characters per word and the average number of words per sentence to make its calculation.^21^
